# Identification of Candidate Genes Associated with Positive and Negative Heterosis in Rice

**DOI:** 10.1371/journal.pone.0095178

**Published:** 2014-04-17

**Authors:** R. C. Venu, Jianbing Ma, Yulin Jia, Guangjie Liu, Melissa H. Jia, Kan Nobuta, M. V. Sreerekha, Karen Moldenhauer, Anna M. McClung, Blake C. Meyers, Guo-Liang Wang

**Affiliations:** 1 Dale Bumpers National Rice Research Center (DB NRRC), Agricultural Research Service, United States Department of Agriculture (USDA-ARS), Stuttgart, Arkansas, United States of America; 2 Rice Research and Extension Center, University of Arkansas Division of Agriculture, Stuttgart, Arkansas, United States of America; 3 Department of Plant Pathology, Ohio State University, Columbus, Ohio, United States of America; 4 Delaware Biotechnology Institute, University of Delaware, Newark, Delaware, United States of America; BASF Plant Sciences – Cropdesign, Belgium

## Abstract

To identify the genes responsible for yield related traits, and heterosis, massively parallel signature sequencing (MPSS) libraries were constructed from leaves, roots and meristem tissues from the two parents, ‘Nipponbare’ and ‘93-11’, and their F_1_ hybrid. From the MPSS libraries, 1–3 million signatures were obtained. Using cluster analysis, commonly and specifically expressed genes in the parents and their F_1_ hybrid were identified. To understand heterosis in the F_1_ hybrid, the differentially expressed genes in the F_1_ hybrid were mapped to yield related quantitative trait loci (QTL) regions using a linkage map constructed from 131 polymorphic simple sequence repeat markers with 266 recombinant inbred lines derived from a cross between Nipponbare and 93-11. QTLs were identified for yield related traits including days to heading, plant height, plant type, number of tillers, main panicle length, number of primary branches per main panicle, number of kernels per main panicle, total kernel weight per main panicle, 1000 grain weight and total grain yield per plant. Seventy one QTLs for these traits were mapped, of which 3 QTLs were novel. Many highly expressed chromatin-related genes in the F_1_ hybrid encoding histone demethylases, histone deacetylases, argonaute-like proteins and polycomb proteins were located in these yield QTL regions. A total of 336 highly expressed transcription factor (TF) genes belonging to 50 TF families were identified in the yield QTL intervals. These findings provide the starting genomic materials to elucidate the molecular basis of yield related traits and heterosis in rice.

## Introduction

Rice is one of the most important cereal crops feeding half of the worlds' population. Because of the increasing population and reduction of arable lands for rice production, improving grain yield is one of the most important goals of rice breeding programs [1], [2]. The genetic basis of yield and its component traits are complex, and controlled simultaneously by QTLs that are sensitive to environmental changes [3]–[5]. Hybrid rice where F_1_ plants are used has provided the highest yield potential in comparison with inbred cultivars. Since the 1970's hybrid rice has been widely cultivated in China and is now being extended to United States and worldwide.

Rice yield is either directly or indirectly affected by various yield related traits including days to heading [DTH], plant height [PHT], lodging [LOG], tiller angle [PTY], numbers of tillers [NOT], number of primary branches per panicle [NOB], total kernel weight per panicle [KWP], number of kernels per panicle [NOK], panicle length [PLE], 1000 grain weight [TGW] and total yield per plant [TYP]. Heading date is important to rice breeders because it affects adaptation of plants to various crop seasons and cultivation areas [6]. Heading date is regulated by a complex gene network consisting of a series of genetic factors [7]. Many genes that control heading date have been identified by QTL analysis [8]–[11]. Some of the important QTLs, *Hd1, Hd3a* and *Ehd1* involved in heading date were cloned [12]–[14]. In addition, genes influencing heading date, plant height and rice yield like *Ghd7* and *Ghd8* were also cloned [15], [16]. A major plant height gene, the semi-dwarf gene *sd1* was responsible for the green revolution in rice [17]. Some major QTLs for grain shape and 1000 grain weight such as *GS3, GW2* and *qSW5/GW5* were fine mapped and cloned [2], [18], [19]. The QTL *Gn1a* influencing the number of kernels per panicle was isolated by a map-based cloning strategy [1]. In addition, QTL controlling grain weight, *gw8.1* and *gw9.1* [20], [21] and number of spikelets per panicle, *GPP1, gpa7* and *SPP3b/TGW3b,* were recently fine mapped [22]–[24]. In spite of hundreds of QTL mapping studies in rice for yield related traits, few of them have been isolated. Most of the genes either cloned or fine mapped so far belong to major QTLs, and the genes located in the minor QTL regions have not been fully explored.

In hybrids, novel patterns of gene action resulting from the combination of allelic variants are thought to be responsible for heterosis [25]–[28]. Dominance [29], over-dominance [30], [31], or epistasis [32], [33] were used to explain heterosis. For example, *indica x japonica* crosses show maximum heterosis compared to any other combination between other subspecies [34]. Gene expression and QTL analysis provide an avenue for identifying candidate genes for heterosis [35]. Several genomic approaches have been employed in rice and many genes underlying yield related traits have been identified [1], [2], [18], [19], [36], [37], [38]. For example, plant height is related to synthesis of sucrose phosphate synthase [SPS] [39], and phytohormones such as gibberellin and brassinolide [40], [41]. Further, large-scale transcriptome profiling has been used to identify the genes related to heterosis in crop plants such as rice [42]–[44], maize [45] and wheat [46]. Using a cDNA microarray consisting of 9198 expressed sequence tags [ESTs], gene expression profiles from an elite hybrid rice Shanyou 63, its parents [Zhenshan 97 and Minghui 63] revealed patterns of expressed genes may be associated with heterosis at three stages of young panicle development [42]. In addition, differentially expressed genes related to heterosis were identified in the super hybrid rice LYP9 compared to its parents [93–11 and PeiAi64S] using microarray and SAGE technologies [44], [45]. The root transcriptomes of the super-hybrid rice variety Xieyou 9308 and its parents were analyzed at tillering and heading stages for identification of candidate genes for heterosis [46] using RNA sequencing technology [RNA-Seq].

Both positive and negative heterosis can be employed in breeding depending on target traits, In general, positive heterosis is desirable for yield, and negative heterosis of growth duration is useful for earliness [47]–[49]. In F_1_ hybrid, the combination of allelic variants results in novel patterns of gene action possibly leading to heterosis [45], [49], [50]. Genetic variation, epistatic interaction, epigenetic modification and small- RNA-directed gene regulation were also shown to be related to heterosis [30], [33], [51]–[53]. Expression of transcription factors [TFs] and polymorphic *cis*-regulatory elements in the promoters of related genes in hybrids play an important role in heterotic gene expression and heterosis in rice [54]. Recently, gene expression profiling in *Arabidopsis* suggests that the genes involved in the circadian rhythm such as MYB-like transcription factors were associated with heterosis [55]. However, the molecular mechanism of either positive or negative heterosis remains poorly understood. For investigating heterosis at the transcriptome level EST library sequencing, microarray hybridization and serial analysis of gene expression [SAGE] have been used in crop plants, However, these technologies have drawbacks, such as low throughput, high cost, low sensitivity, cloning bias, high background signal, and pre-determined probe requirements [43], [44], [56]. Deep sequencing technologies including llumina's Massively Parallel Signature Sequencing [MPSS], Sequencing By Synthesis [SBS], RNA-Seq and pyrosequencing which offer large sequencing output with lower cost, have been widely applied in studying transcriptomes in plants and animals [47], [57]. The MPSS and SBS tags are short cDNA tags or digital gene expression tags, which are mainly derived from the 3′ regions of a transcript. These are deep sequencing methods previously used in rice and Arabidopsis [58], [59]. These tag- or sequence-based technologies determine the expression level of a gene by counting the precise abundance of a specific transcript in a library [25], [58], [59].

An inter-subspecific F1 hybrid was developed from a cross between Nipponbare [*japonica*] and 93-11[*indica*]. The genomes of both parents were completely sequenced [60]–[62]. Nipponbare is a rice cultivar developed in Japan [63]. Cultivar 93-11 is an elite parental line used in developing several super hybrid rice such as LYP9, YLY7, YLY1 etc. in China [44]. Thus, the F_1_ hybrid produced in this study provides a unique opportunity to investigate the molecular basis of yield related traits. The objectives of the present study were to 1) evaluate yield related traits and determine the transcription profiles of leaves, roots and meristems of the two parents, Nipponbare and 93-11] and their F_1_ hybrids using MPSS technology; 2) determine their commonly and specifically expressed genes; and 3) to map differentially expressed transcripts onto a genetic map and analyze their potentials for rice heterosis.

## Results

### Phenotyping and transcriptome sequencing of hybrid and their parents

F_1_ hybrid plants, RILs and the parents Nipponbare and 93-11 were phenotyped for yield traits (Table 1, Table S1). The F_1_ hybrid showed longer DTH [124 days], increased PHT [134 cm], narrower PTY [1], increased NOT [48], slightly shorter PLE [20.5], decreased number of NOK [16], decreased KWP [0.42], intermediate TGW [26.4] and decreased TYP [5.9] compared to the inbred parents (Figure S1). In this study, DTH, PHT and NOT in the F_1_ hybrid plants were greater than in the parents. PTY, PLE, NOK, KWP, and TYP in the F_1_ hybrid were less than the parents.

**Table 1:** Summary statistics of phenotypic performance of recombinant inbred lines, F_1_ hybrids and their parents Nipponbare and 93-11 (Parenthesis indicate averages).

Trait	Stuttgart, AR-2009			Stuttgart, AR-2010			Stuttgart, AR-2011				Stuttgart, AR-2012			Beaumont, TX-2009			Beaumont, TX-2010		
	NPB	93-11	RILs	NPB	93-11	RILs	NPB	93-11	RILs	F_1_ hybrids	NPB	93-11	RILs	NPB	93-11	RILs	NPB	93-11	RILs
**Number of days to heading**	84–87 (85.5)	86–88 (87)	68–131 (85.8)	90–91 (90.3)	84–86 (85.2)	63.6-117-6 (80.1)	96–99 (97.7)	74–78 (75.2)	61.7–105 (72.8)	124	88.3–91.7 (90.1)	92.7–102 (96.36)	68–111.6 (83.6)	80–81 (79.8)	85–86 (85.5)	67–137 (92.2)	79 (79)	87–100 (94.3)	69.5–111.5 (87.8)
**Plant height (cm)**	86–103 (93.5)	99–120 (111.4)	60–168 (106.9)	88–98 (93.3)	124–136 (130)	67.33–152 (108.6)	70–78 (73.3)	118–128 (123.1)	64–154 (104.3)	134	84–94 (90)	98–118 (108)	54–146 (102.6)	79.5–82.5 (82)	100.5–105-5 (103)	66.7–153.7 (98.5)	70–84 (76)	84–90 (86.8)	51.88–128.6 (83.4)
**Lodging status**	0 (0)	0 (0)	0–8 (0.3)	0 (0)	0 (0)	0–3 (0.03)	-	-	-	-	-	-	-	0	0	0–3 (0.04)	0	0	0–0.33 (0.002)
**Tiller angle (plant type)**	3 (3)	3 (3)	1–7 (3.8)	3 (3)	3 (3)	1–7 (3.4)	3 (3)	3 (3)	1–7 (3.8)	1	3 (3)	3(3)	1–6 (3.1)	1–3 (2)	3–5 (4)	1–7 (2.8)	1–3 (2)	3 (3)	1–7 (2.9)
**Number of tillers per plant**	21–29 (25.1)	18–25.3 (21.8)	8.3–81 (30.6)	-	-	-	27–41 (38.4)	29–44 (36.5)	22–62 (36.6)	48	19–34.3 (25.8)	18–23 (19.75)	10.5–48.4 (26.8)	29–42 (37.7)	35–39 (36.7)	15.7–70.7 (37.2)	-	-	-
**Main panicle length (cm)**	21.3–22.2 (21.9)	24–25 (24)	14.3–32.5 (22.2)	-	-	-	20–22 (21.5)	22–24 (22.5)	15.7–29.2 (21.3)	20.5	20–23.2 (21.7)	23.8–26.5 (25)	15.6–31 (23)	20–21 (20.4)	24.5–26.7 (25.7)	16.5–32.5 (22.4)	-	-	-
**Number of branches in main panicle**	9.7–11 (10.3)	10–12.3 (11.1)	7–15.7 (10.6)	-	-	-	-	-	-	-	8.7–10.3 (9.5)	10–12 (11)	6.5–17.5 (10)	10–12 (11)	10–12 (11.33)	8–18 (11.6)	-	-	-
**Number of grains per main panicle**	92.7–113.3 (105.8)	165.3–191 (179.3)	10–272.7 (126.2)	-	-	-	27–41 (35)	152–192 (177.9)	2.8–176.4 (76.9)	16	98.3–138 (117.2)	68.3–230 (160.2)	19.5–305.6 (112.5)	93–105 (99.3)	180–201 (189)	28.7–248.7 (114.9)	-	-	-
**Total kernel weight per panicle**	2.6–3.1 (2.93)	5.8–6.2 (6)	0.2–7.4 (3.6)	-	-	-	0.9–1.1 (1.05)	5.1–5.5 (5.2)	0.067–5 (1.97)	0.42	2–3.5 (2.8)	3.3–6.6 (5.4)	0.4–7 (2.8)	2.4–2.6 (2.5)	5.2–5.8 (5.4)	1.1–5.4 (3.08)	-	-	-
**1000 grain weight (grams)**	27.1–28.1 (27.8)	32.5–34.8 (33.4)	11.1–43.4 (27.8)	-	-	-	24–29 (26)	26.8–29.3 (28.2)	15.9–39.7 (25)	26.4	24.2–25.7 (25.3)	25.4–28.7 (27)	16.5–40.2 (25.2)	25–26 (24.8)	28–29 (28.8)	15.2–33.2 (24.8)	-	-	-
**Total grain yield per plant (grams)**	50.1–65.1 (56.6)	97.2–126.1 (107.9)	6–179.5 (74.5)	-	-	-	30–46 (40.2)	152–187 (155.6)	3.9–155 (55)	5.9	52.3–89.3 (71)	78.6 (78.6)	13.6–223.2 (79.8)	52–73 (69.6)	140–156 (152)	27.3–149.5 (82.9)	-	-	-

About 1.0 to 3.0 million 17-base MPSS signatures were obtained in the 21 libraries (Table 2, Table S2). These signatures were clustered and processed with reliability and significance filters as described by Meyers et al. [40]–[42] (Figure S2). To compare the expression levels across the libraries, the frequency of signatures in the individual libraries were normalized to one million [transcripts per million or TPM] [40]–[42]. The number of distinct signatures ranged from 14,127 to 28,621 in the MPSS libraries. The number of distinct genes identified using reliable and significance filtered signatures from 5,444 to 12,717 genes. About 56 to 87% of the signatures from Nipponbare matched to the Nipponbare genomic sequence. Similarly, about 77 to 87% of the signatures obtained from the 93-11 tissues matched to the 93-11 genomic sequence (Table S2). In F1, about 74 to 84% of signatures matched Nipponbare and 75 to 85% of signatures matched 93-11 (Table S2). The significant MPSS signatures from all 21 libraries were classified into seven classes based on their location on the annotated genes (Table S3).

**Table 2:** Characteristics of MPSS libraries of Nipponbare, 93-11 and F_1_ hybrid from leaves, roots and meristem tissues.

Category	Nipponbare			93-11			F_1_ hybrid		
	Leaves∞	Roots∞	Meristems	Leaves∞	Roots∞	Meristems	Leaves∞	Roots∞	Meristems
Total reads sequenced	4940921	5293337	2,568,641	4902516	4319104	2,112,790	4687682	4642271	3,045,290
Distinct	60782	48206	28621	38478	34771	21836	44455	29119	24715
Reliable*	44971	38064	24183	31696	28109	17570	38082	24291	19945
Significant*	48688	38330	24305	33332	29195	19027	40037	24476	20056
Reliable and Significant (≥4 TPM)	35617	27282	18106	26697	22996	14081	32370	17289	15033
1–100 TPM	57321	45257	26237	35346	31371	19695	41236	26983	22579
101–1,000 TPM	3279	2804	2245	2973	3249	1962	3072	1958	1950
1,001–10,000 TPM	190	151	134	169	158	166	161	184	176
>10,000 TPM	8	4	5	6	3	13	7	10	10
#Distinct genes	11902	11816	9360	10890	10186	6696	12717	8007	7591
$ Genome match	22786 (64%)	21122 (77%)	14936 (83%)	20991 (79%)	17756 (77%)	10777 (77%)	24717(76%) *24895* (*77%)*	12855(74%) *13005* (*75%)*	11763 (78%) *11857* (*79%)*

Note: *- Number of signatures passed through reliability and significance filters as described by Meyers et al. [58]–[59]

#- Using all reliable and significance filter signatures.

$- Number and % of signatures match Nipponbare/93-11/both genomic sequences (≥4 TPM).

∞ - Mean calculated from four leaf libraries are shown for leaf libraries. Similarly, the mean calculated from two root libraries are shown for root libraries (details of all the libraries can be found in Table S4).

Italics indicate- match to 93-11 genomic sequence.

### Commonly and specifically expressed genes

**Figure 1:**
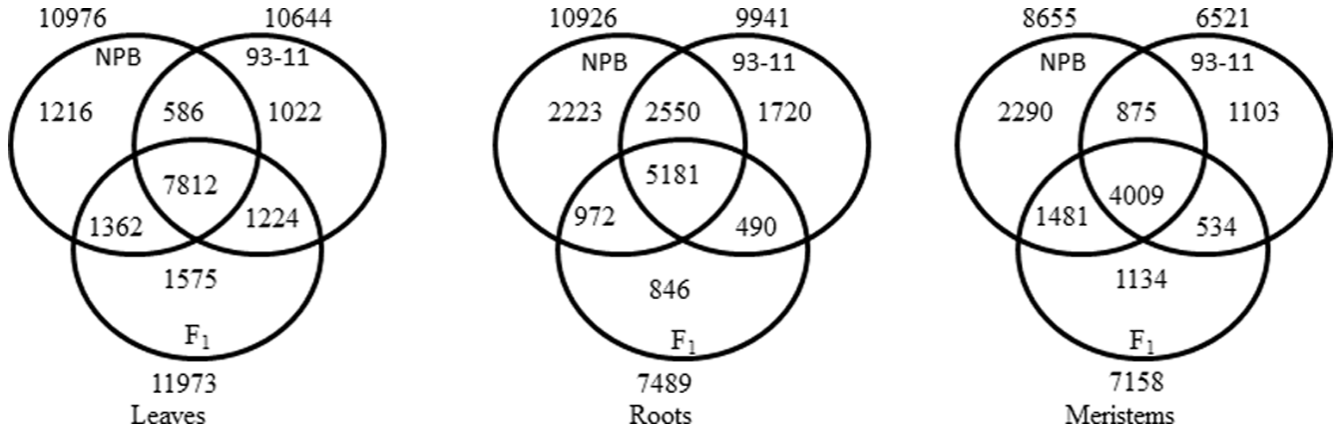
Identification of commonly and specifically expressed genes in Nipponbare, 93-11 and F_1_ hybrid tissues. Signatures matched to both Nipponbare and 93-11 genomes were used to avoid sequence bias in the identification of commonly and specifically expressed genes among Nipponbare, 93-11 and F_1_ hybrid.

Based on the results of a Venn diagram, we analyzed the differences that existed in gene expression among leaves, roots and meristem tissues. The similarities between any two genotypes [Nipponbare, 93-11 and F_1_ hybrid] were established based on the number of similar genes expressed between any two genotypes, and the number of genotype specifically expressed genes. The number of genes expressed in all three tissues [leaves, roots and meristems] in Nipponbare, 93-11 and the F_1_ hybrid were identified. A total of 7,812, 5,181 and 4,009 genes were commonly expressed in leaves, roots and meristem tissues, respectively. The number of commonly expressed genes was higher than specifically expressed genes in all 3 tissues (Figure 1).

**Figure 2:**
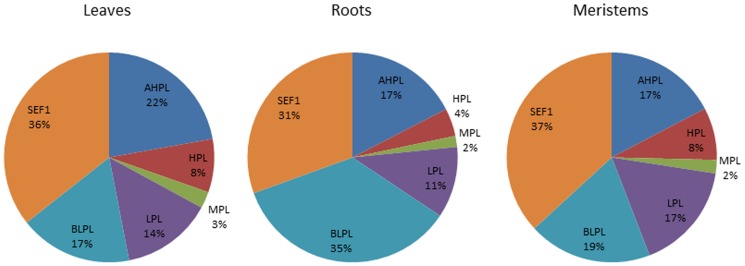
Distribution of ‘gene expression level categories’ in F1 hybrid's leaf, root and meristem tissues. The gene expression levels in leaf, root and meristem tissues of F_1_ hybrid were measured in comparison to their parents Nipponbare and 93-11. The differentially expressed signatures in F_1_ hybrid belonging to expression patterns: above high parent level (AHPL), high parent level (HPL), mid parent level (MPL), low parent level (LPL), below low parent level (BLPL) and transcripts expressed specifically in F_1_ and absent in parents (SEF_1_) are represented here.

The expression levels of Nipponbare and 93-11 were compared with their F_1_ hybrid and the differentially expressed signatures were classified into eight expression patterns: above high parent level [AHPL], high parent level [HPL], mid parent level [MPL], low parent level [LPL], below low parent level [BLPL], transcripts specifically expressed in F_1_ and absent in parents [SEF_1_], transcripts expressed in either one of the parents and F_1_ [EOPF_1_], transcripts expressed in either of the parents but absent in F_1_ [EPAF_1_]. To observe which gene expression patterns were highly represented in the F_1_ hybrid, the gene expression patterns, including AHPL, HPL, MPL, LPL, BLPL and SEF1 were compared in leaves, roots and meristem tissues (Table S4). AHPL and SEF_1_ represent a majority of the gene expression pattern in leaves, roots and meristems (Figure 2). In leaves, roots and meristems combined AHPL and SEF1 expression patterns represented 58%, 48% and 54%, respectively. AHPL expression patterns represented 22%, 17%, and 17% while SEF1 expression patterns represented 36%, 31% and 37% in leaves, roots and meristems, respectively. These findings suggest that novel patterns of gene action thought to be involved in heterosis resulting in allelic variants from the parents and the hybrid.

### Mapping differentially expressed genes

To better understand mechanisms underlying positive and negative heterosis in F_1_ hybrids, a linkage map was constructed using 131 polymorphic simple sequence repeat [SSR] markers with 266 RILs derived from a cross between Nipponbare and 93-11. Phenotypes of yield related traits were evaluated in 6 field studies [Stuttgart-2009, Stuttgart-2010, Stuttgart-2011, Stuttgart-2012, Beaumont-2009 and Beaumont-2010] (Table 1). Transgressive segregation was observed among the RILs for yield related traits (Table 1). Obvious transgressive segregation was observed for PTY [1]–[7], LOG [0–8], PHT [60–168 cm], NOT [8.3–81], NOK [10–272.7], TGW [11.1–43.4 g] and TYP [6–179.5 g] compared to other traits evaluated (Table 1; Figure S1; Table S1).

**Figure 3:**
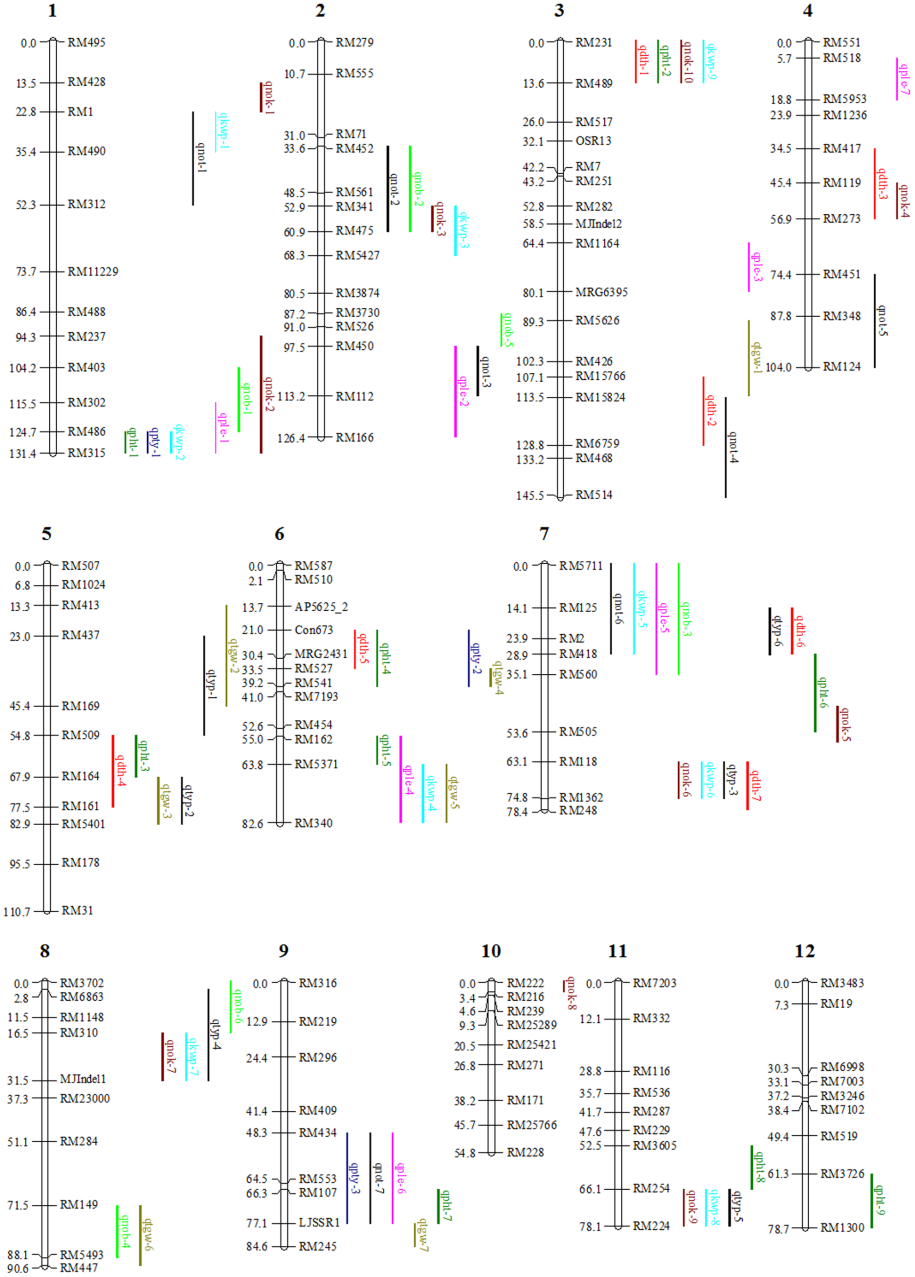
Chromosomal locations of yield related QTL identified using RILs obtained from Nipponbare x 93-11 cross. Seventy one QTL identified were mapped for yield related traits including days to heading (*qdth*), plant height (*qpht*), tiller angle (*qpty*), tiller number (*qnot*), panicle length (*qple*), number of primary branches per panicle (*qnob*), number of kernels per panicle (*qnok*), total kernel weight per panicle (*qkwp*), 1000 grain weight (*qtgw*) and total grain yield per plant (*qtyp*).

Seventy-one QTLs above the significant threshold level for yield related traits were mapped (Figure 3; Table 3; Table S5). A total of three novel QTLs related to PTY [2] and KWP [1] were identified. No QTL above the significant threshold level was found for LOG. We selected the QTLs which showed mean LOD scores of ≥5 for a given trait for identification of differentially expressed genes located in the 71 mapped QTLs (Table S6). Based on the physical location of SSR markers on the chromosomes, the differentially expressed genes between flanking markers were identified. Expression patterns for the differentially expressed genes in a particular QTL were listed in Table S6 (Table S6).

**Table 3:** List of previously published QTLs for yield and yield related traits identified in recombinant inbred line population (Nipponbare x 93-11) based on Gramene QTL database.

Trait	Experiment location	Marker interval	Genetic distance (cM)	LOD peak position	Ch.	R^2^ (%)	Additive effect∧	LOD threshold	LOD value	Avg. LOD score	No. of genes present	QTL name in this study	Published QTL
Days to heading													
	2009-Beaumont, TX	RM231–RM489	0–13.6	0.2	3	12.7	−4.0	3.6	5.8	7.6	176	*qdth-1* ^#^	*QTL3, dth3.1, qFDN-3*
	2009-Stuttgart, AR	RM231–RM489	0–13.6	0	3	10.2	−3.0	3.6	11.2				
	2010-Stuttgart, AR	RM231–RM489	0–13.6	4	3	11.3	−3.5	3.6	10.5				
	2010-Beaumont, TX	RM231–RM489	0–13.6	5	3	9.6	−2.8	3.5	7.5				
	2011-Stuttgart, AR	RM231–RM489	0–13.6	1	3	7.1	−2.4	3.8	4.9				
	2012-Stuttgart, AR	RM231–RM489	0–13.6	2.1	3	8.13	−2.8	3.5	5.53				
	2009-Stuttgart, AR	RM15766–RM15824	107.2–113.5	108.6	3	3.3	−1.7	3.6	3.5	6.1	321	*qdth-2* ^#^	*QHd3c, Hd6, QHd3b*
	2010-Beaumont, TX	RM15824–RM6759	113.5–128.8	122.6	3	12.4	−3.1	3.5	8.7				
	2010-Stuttgart, AR	Con673–RM527	21–33.5	31.4	6	49.9	11.3	3.6	43.2	25.0	126	*qdth-5* ^#^	*Hd1, Hd-1*
	2009-Beaumont, TX	Con673–RM527	21–33.5	30.5	6	21.0	8.6	3.6	8.4				
	2010-Beaumont, TX	MRG2431–RM527	30.4–33.5	31.3	6	17.4	5.7	3.5	15.7				
	2009-Stuttgart, AR	MRG2431–RM527	30.4–33.5	31.5	6	49.9	9.9	3.6	43.5				
	2011-Stuttgart, AR	Con673–MRG2431	21–30.4	29.1	6	40.7	9.9	3.8	23.7				
	2012-Stuttgar, AR	Con673–RM527	21–33.5	29	6	25.2	7.5	3.5	15.4				
	2010-Beaumont, TX	RM125–RM418	14.1–28.9	23	7	10.4	−2.9	3.4	9.4	9.4	359	*qdth-6*	*hd7c, Hd7, qHDD7-1, Hd4, qHD-7*
	2009-Stuttgart, AR	RM1362–RM248	74.8–78.4	78	7	3.0	1.7	3.6	3.4	5.5	41	*qdth-7* ^#^	*qDTH-7, QTL7b*
	2010-Stuttgart, AR	RM1362–RM248	74.8–78.4	78	7	7.4	2.9	3.6	8.1				
	2009-Beaumont, TX	RM1362–RM248	74.8–78.4	78	7	7.5	3.1	3.6	3.6				
	2011-Stuttgart, AR	RM118–RM248	63.1–78.4	78	7	8.3	2.7	3.8	6.0				
	2012-Stuttgart, AR	RM1362-RM248	74.8–78.4	78	7	8.4	2.9	3.5	6.3				
**Plant height**													
	2009-Beaumont, TX	RM486–RM315	124.7–131.4	130.6	1	22.8	8.8	3.8	7.4	16.9	147	*qpht-1* ^#^	*Ph1, qph1.4, qPHT-1*
	2009-Stuttgart, AR	RM486–RM315	124.7–131.4	131	1	32.0	12.4	3.5	21.4				
	2010-Stuttgart, AR	RM486–RM315	124.7–131.4	130.6	1	29.2	9.7	3.6	22.1				
	2010-Beaumont, TX	RM486–RM315	124.7–131.4	130.6	1	21.7	7.2	3.4	17.8				
	2011-Stuttgart, AR	RM486–RM315	124.7–131.4	130.6	1	28.6	9.3	3.7	19.1				
	2012-Stuttgart, AR	RM486–RM315	124.7–131.4	130.6	1	21.0	8.4	3.4	13.4				
	2011-Stuttgart, AR	RM162–RM5371	55–63.8	62	6	7.0	−5.3	3.7	5.0	5.0	81	*qpht-5*	*Fh6-2, ph6.1, QPh6*
	2010-Beaumont, TX	RM107–LJSSR1	66.3–77.1	69.4	9	7.7	4.2	3.4	6.1	5.1	137	*qpht-7* ^#^	*Fh9-2*
	2009-Stuttgart, AR	RM107–LJSSR1	66.3–77.1	71.4	9	4.6	4.4	3.5	4.1				
**Tiller angle**													
	2010-Stuttgart, AR	RM434–LJSSR1	48.3–77.1	70.2	9	48.6	−1.1	3.5	36.6	31.80	477	*qpty-3* ^#^	*ta9, Ta, qTA-9a, ta9.1*
	2009-Beaumont, TX	RM434–LJSSR1	48.3–77.1	66.3	9	37.9	−1.6	4	13.2				
	2010-Beaumont, TX	RM107–LJSSR1	66.3–77.1	69.6	9	58.8	−1.2	3.6	45.6				
	2009-Stuttgart, AR	RM107–LJSSR1	66.3–77.1	69.7	9	43.0	−1.3	3.4	32.7				
	2011-Stuttgart, AR	RM434–LJSSR1	48.3–77.1	61.6	9	50.9	−1.5	3.7	25.2				
	2012-Stuttgart, AR	RM107–LJSSR1	66.3–77.1	70.5	9	66.7	−1.4	3.6	37.56				
**Tiller number**													
	2010-Beaumont, TX	RM452–RM475	33.6–60.9	46.6	2	8.3	2.9	3.5	6.4	6.4	410	*qnot-2*	*qNOT2-1, mtn2-1, tn2-1, qNOT2-1*
	2009-Beaumont, TX	RM15824–RM514	113.5–145.5	131	3	13.7	−4.0	3.9	5.0	5.0	583	*qnot-4*	*tn3-4*
	2010-Beaumont, TX	RM451–RM124	74.4–104	82.2	4	9.5	−3.0	3.5	6.8	6.1	541	*qnot-5* ^#^	*tp4, mtn4, tn4-3*
	2011-Stuttgart, AR	RM348–RM124	87.8–104	99.5	4	10.0	−2.2	3.5	5.4				
	2010-Beaumont, TX	RM125–RM418	14.1–28.9	18.9	7	7.9	2.8	3.5	5.8	5.1	515	*qnot-6* ^#^	*tp7, tn7.1, qTN-7-2*
	2011-Stuttgart, AR	RM5711–RM125	0–14.1	10.9	7	8.2	2.0	3.5	4.5				
	2010-Beaumont, TX	RM434–LJSSR1	48.3–77.1	70.8	9	9.7	−3.1	3.5	7.3	6.3	477	*qnot-7* ^#^	*qNOT9-1*
	2011-Stuttgart, AR	RM434–LJSSR1	48.3–77.1	67.2	9	9.2	−2.2	3.5	5.9				
	2012-Stuttgart, AR	RM107–LJSSR1	66.3–77.1	74.3	9	9.4	−2.1	3.6	5.6				
**Panicle length**													
	2009-Beaumont, TX	RM302–RM315	115.6–131.4	131	1	16.9	1.2	3.6	5.1	5.1	302	*qple-1*	*pI1.1, qPL-1*
	2011-Stuttgart, AR	RM450–RM166	97.5–126.4	115.2	2	7.7	−0.7	3.6	5.1	5.1	504	*qple-2*	*pI2a*
	2009-Stuttgart, AR	RM5711–RM2	0–23.9	16.3	7	7.9	−0.8	3.5	5.3	6.7	515	*qple-5* ^#^	*QPI7, pI7.1*
	2011-Stuttgart, AR	RM2–RM560	23.9–35.1	28.9	7	10.4	−0.7	3.6	8.1				
**Number of branches**													
	2009-Stuttgart, AR	RM452–RM475	33.6–60.9	44.6	2	11.3	−0.5	3.5	6.3	6.3	410	*qnob-2*	*qNRB-2-1*
	2009-Beaumont, TX	RM149–RM5493	71.5–88.1	84.5	8	21.0	1.0	4.1	5.5	5.5	116	*qnob-4*	*qNRB-8-1, qNRB-8-2*
**Number of grains per panicle**													
	2009-Stuttgart, AR	RM341–RM475	52.9–60.9	62.2	2	8.6	−13.3	3.8	6.5	6.5	54	*qnok-3*	*gn2.1, sn2.1*
**Total grain wt. per panicle**													
	2009-Stuttgart, AR	RM486–RM315	124.7–131.4	130.7	1	8.5	0.3	3.6	6.6	6.6	147	*qkwp-2*	*Pdw1-1*
	2009-Stuttgart, AR	RM341–RM5427	52.9–68.3	61.1	2	7.0	−0.3	3.6	5.6	5.6	122	*qkwp-3*	*Gw2*
	2009-Beaumont, TX	RM5711–RM2	0–23.9	17.2	7	16.6	−0.4	3.8	5.5	5.5	515	*qkwp-5* ^#^	*Pdw7*
	2009-Stuttgart, AR	RM125–RM418	14.1–28.9	20.3	7	9.1	−0.4	3.6	6.0				
	2012-Stuttgart, AR	RM2–RM418	23.9–28.9	26.1	7	7.7	−0.3	3.3	5.0				
	2009-Beaumont, TX	RM254–RM224	66.1–78.1	77.2	11	14.4	−0.4	3.8	5.6	5.6	124	*qkwp-8*	Unnamed*
**1000 grain wt.**													
	2009-Stuttgart, AR	RM413–RM169	13.3–45.4	14.5	5	9.2	1.3	3.7	7.8	6.5	279	*qtgw-2* ^#^	*gw5a, gw5*
	2009-Beaumont, TX	RM437–RM169	23–45.4	28.6	5	17.7	1.5	3.6	4.8				
	2011-Stuttgart, AR	RM437–RM169	23–45.4	29.6	5	13.5	1.4	3.4	5.6				
	2012-Stuttgart, AR	RM437–RM169	23–45.4	28.6	5	15.3	1.3	3.7	7.8				
	2009-Stuttgart, AR	RM5371–RM340	63.8–82.6	80.7	6	8.0	−1.1	3.7	6.3	6.3	190	*qtgw-5*	*qGW-6, gw6c, gw6b*
**Grain yield per plant**													
	2009-Beaumont, TX	RM6863–RM310	2.8–16.5	11.6	8	14.1	−10.4	3.5	5.4	5.7	442	*qtyp-4* ^#^	*qYI-8, yld8.3*
	2011-Stuttgart, AR	RM310–MJIndel1	16.5–31.5	23	8	14.2	−9.5	3.8	6.1				

Number of expressed genes identified in leaves, roots and meristem tissues based on their location in the mapped yield and yield related QTLs. LOD score ≥5 was used to identify the genes located in the mapped yield and yield related QTLs. Average LOD score was calculated for each QTL detected based on 6 experiments (more details are given in the supplemental table 1 and supplemental table 5).

Note: *- indicate the QTL is unnamed in Gramene rice QTL database-http://gramene.org/qtl/ or Japanese Rice QTL database - http://qtaro.abr.affrc.go.jp/.

#-Qtl identified in multiple locations.

∧- A positive additive effect indicates the Nipponbare allele increases the phenotype.

Of the seven QTLs for DTH, five showed average LOD score ≥5. The QTL *qdth5* located at 21–33.5 cM on chromosome 6 [close to the flanking markers Con673 and RM527] had the highest mean LOD score of 25. Nine QTL were identified for PHT, of which three showed an average LOD score of ≥5. The QTL *qpht1* located at 124.7–131.4 cM on chromosome 1 [close to the flanking markers RM486 and RM315] showed a mean LOD score of 16.8. Both parents Nipponbare and 93-11 have intermediate tiller angle [about 45°], but the F_1_ hybrid has erect tillers and an angle less than 30° from the perpendicular. Three QTLs were detected for tiller angle, of which *qpty1* and *qpty2* were not reported in gramene [ftp://ftp.gramene.org/pub/gramene/CURRENT_RELEASE/data/qtl/] and Japanese database [http://qtaro.abr.affrc.go.jp/qtab/table]. The *qpty3* allele located on chromosome 9 contained 477 differentially expressed genes between marker intervals [RM434-LJSSR1] and was detected in all six field experiments with a mean LOD score of 31.8. Seven QTL have been identified for NOT, of which five showed mean LOD scores of ≥5. The QTLs *qnot5* [chromosome 4; 74.4–104 cM] and *qnot7* [chromosome 9; 48.3–77.1 cM] showed a mean LOD score of 6.1 and 6.3, respectively.

We identified seven QTLs related for PLE, of which three showed average LOD scores of ≥5. We identified six QTLs related to NOB, two of which showed an average LOD score of ≥5. A total of ten QTLs have been identified for NOK. Of these QTL only *qnok3* showed a significant LOD score of 5 or higher (LOD 6.5). Of the nine QTLs detected for Total kernel weight per panicle, *qkwp7* was a novel QTL. The allele *qkwp8* was detected in the public rice QTL database, but was unnamed. QTLs for KWP were found on chromosomes 1, 2, 6, 7, 8 and 11 and four of these had a LOD ≥5. A total of seven QTLs were detected for TGW. The *qtgw1, qtgw3, qtgw5 and qtgw7* alleles were only detected at the Stuttgart location. Of these, two QTLs had a LOD ≥5. Total grain yield per plant is the most important trait to be mapped on chromosomes for improving rice yield. The QTLs related to TYP were detected in the Beaumont 2009 and Stuttgart 2011. Six QTLs were found to be associated with TYP, of which four QTLs [*qtyp1, qtyp2, qtyp4* and *qtyp5*] were detected at the Beaumont 2009 location. The QTL *qtyp4* located on chromosome 8 at 2.8–31.5 cM showed a LOD score of 5.72, and contained 442 differentially expressed genes between the closely linked markers RM6863 and MJIndel1.

Expressed TF genes located in the mapped yield QTL were identified using homology search in the rice transcription factor database [http://ricetfdb.bio.uni-potsdam.de/v2.1/]. A total of 336 expressed TF genes belonging to 50 TF families, were identified in combined leaves, roots and meristem tissues, located in yield related QTL intervals (Table S7). Six TF genes were expressed at AHPL in all three tissues. This included three genes encoding helix-loop-helix [HLH] DNA binding domain containing proteins Os03g53020 [*qdth2, qnot4*], Os01g57580 [*qple1*] and Os02g47660 [*qple2*]. In addition, a ZIM motif family protein [Os03g08330], gibberellins response modulator gene belonging to GRAS TF family [Os03g49990], and homeobox associated leucine zipper family protein belonging to HB TF family [Os09g29460] located at mapped yield related QTL regions, showed the expression pattern AHPL in all three tissues-leaves, roots and meristems. Three TF genes showing expression pattern BLPL in all three tissues included a gene encoding homeobox domain containing protein belonging to HB TF family [Os02g49700; *qple2*], a transcriptional adaptor gene belonging to MYB TF family [Os03g53960; *qnot4, qdth2*], and a zinc-finger protein 1 gene belong to C2H2 TF family [Os03g55540; *qnot4, qdth2*] (Table S7).

Some of the highly expressed TF genes showing the AHPL expression pattern in both leaves and roots included: gene encoding for protein PHD finger protein gene [Os02g35600; *qkwp3*], gene encoding for ULTRAPETALA2 protein [Os01g57240; *qple1*], gene encoding auxin response factor 1 [Os02g35140; *qkwp3*] and gene encoding bZIP transcription factor [Os02g52780; *qple2*], gene encoding expressed protein [Os04g50120; *qnot5*] and, gene encoding WRKY DNA binding domain containing protein [Os04g51560; *qnot5*]. Some TF genes showed AHPL expression pattern in both leaves and meristems include gene encoding homeobox protein knotted-1-like 3 gene [Os06g43860; *qtgw5*], gene encoding AP2 domain containing protein [Os04g55520; *qnot5*], gene encoding RNA recognition motif family protein [Os07g48410; *qdth7*] and gene encoding *myb* like DNA binding domain protein [Os03g55590; *qnot4*]. TF genes showed AHPL expression pattern in both roots and meristems include gene encoding zinc finger protein [Os03g60570; *qnot4*], gene encoding DREB1A protein [Os09g35030; *qpht7*] and gene encoding GRAS family protein [Os04g50060; *qnot5*] (Table S7). In leaves, roots and meristems the highly expressed TF genes belonging to AHPL expression pattern encoding SET domain containing protein [Os02g50100, *qple2*], TF TGA4 protein [Os08g07970; *qtyp4*; bZIP family] and Myb-like DNA binding domain protein [Os09g31454; *qpty3 or qnot7*] respectively were identified.

### Expression of genes involved in epigenetics

A total of 99 epigenetic/chromatin-related genes were expressed in leaves, roots and meristem tissues (Table S8). Several genes belonging to flowering, histone demethylases, histone deacetylases, genes encoding argonaute like proteins and polycomb group genes were highly represented. In leaves, genes belonging to the AHPL expression pattern were expressed including genes encoding flowering control-associated proteins [Os03g58070- *qnot4*; Os02g49230-*qple2*; Os06g15330-*qdth5*; Os06g44450-*qtgw5*; Os02g49840-*qple2*; Os06g16370-*qdth5*; Os03g50310-*qdth2*], genes encoding methyl binding domain proteins [Os04g52380-*qnot5*; Os09g29750-*qpty3,qnot7*], polycomb group gene [Os08g04270-q*pty4*]; jumonji domain group gene [Os05g10770-*qtgw2*], a gene encoding an argonaute-like protein [Os07g28850-*qnot6, qple5, qkwp5, qdth6*], piwi domain containing gene [Os03g57560-*qnot4*], a gene with a double stranded RNA-binding motif [Os05g05790-*qtgw2*], histone deacetylases [Os07g06980-*qnot6, qple5, qkwp5*], histone demethylases [Os08g04780 -*qtyp4*], histone H1 linker protein [Os03g58470 -*qnot4*], RNA helicases [Os03g06440-*qdth1*] and genes encoding bromodomain containing proteins [Os08g09340-*qtyp4*, Os09g33980-*qpty3, qnot7, qpht7*]. In roots, some of the genes belonging to the AHPL expression pattern were expressed including: genes encoding a histone H1 linker protein [Os03g58470-*qnot4*], bromodomain-containing proteins [Os08g39980-*qnob4*, Os08g09340-*qtyp4*], double-stranded RNA binding motif family protein [Os05g05790-*qtgw2*], histone ubiquitination proteins [group B Rad6 homolog] [Os03g57790-*qnot4*] and flowering control-associated protein [Os02g52340 -*qple2*] (Table S8).

### Functional classification of genes showing AHPL expression pattern in F_1_ hybrids

The genes in the mapped yield related QTL showing AHPL expression patterns were classified into different groups based on KEGG's functional classification of genes (Figure S3). Biochemical pathways including carbohydrate metabolism, energy metabolism and metabolism of cofactors and vitamins were highly represented in leaves, roots and meristem tissues. Leaf and meristem tissues showed expression of more energy and nucleotide metabolism genes compared to roots (Figure S3). Because in leaves, seven biochemical pathways including carbohydrate metabolism, energy metabolism, nucleotide metabolism, metabolism of cofactors and vitamins, amino acid metabolism, translation, and sorting and degradation were highly represented, we further classified these genes based on mapped QTLs. More carbohydrate metabolism related genes were present in QTLs *qdth2, qnot4, qnot6, qple5* and *qkwp5*. The QTLs *qnot4* and *qnot5* were highly represented by genes belonging to all seven biochemical pathways (Figure S3). Some of the genes belonging to the photosynthesis and carbon fixation [dark cycle/Calvin cycle] pathways showed AHPL expression patterns in leaves included the genes encoding ribulose-phosphate 3-epimerase [Os09g32810- *qpty3* or *qnot7*], uridine/cytidine kinase-like 1 [Os09g32820- *qpty3* or *qnot7*], ribose-5-phosphate isomerase [Os03g56869- *qnot4*], an expressed protein [Os03g56860- *qnot4*], vacuolar ATP synthase subunit D 1 [Os04g55040-*qnot5*], vacuolar ATP synthase catalytic subunit A [Os06g45120-*tgw5*], ferredoxin [Os09g33950-*qpht7, qpty3, qnot7*] and ferredoxin-NADP reductase [Os03g57120-*qnot4*]. The genes encoding phosphoglucomutase [Os03g50480-*qdth2*] and sucrose-phosphate synthase 1 [Os08g20660- *qtyp4*] (Table S6), belonging to sucrose biosynthesis also showed an AHPL expression pattern in leaves.

### Mapped QTL regions covering previously cloned yield related genes

The gene for grain number *Gn1a* [Os01g56810; cytokinin dehydrogenase 5 precursor] was located in the QTL for PLE [*qple1*]. The gene for panicle number, panicle branching and high grain productivity was encoded by *OsSPL14* [squamosa promoter-binding-like protein 9] and located in this study at the QTL for NOB [*qnob4*]. The gene for DTH, *Hd1* [zinc finger protein CONSTANS] was located at the QTL *qdth5*. The gene for PTY *TAC1*, an expressed protein gene, was located at the QTL region for PTY/NOT/PHT [*qpty3/qnot7/qpht7*]. The gene for grain width and weight *GW2*, which showed homology to gene Os02g53140 [ubiquitin ligase protein COP1] was located in the QTL *qple2*. Similarly, another homologous gene Os02g19140 [ubiquitin ligase SINAT4] was located in the QTL *qnot2/qnob2*. The gene for dense panicle, high grain number per panicle and erect panicle, *DEP1* [Os09g26999-keratin-associated protein 5-4] was located in the QTL *qpty3/qnot7*. Many of these genes were expressed in either of the parents and/or the F_1_ (Table S9).

## Discussion

For the first time, genome-wide gene expression from the leaves, roots and meristems of rice were mapped onto 71 QTLs of yield related traits. Among them, sixty eight QTLs had have been previously reported by others; while, three QTLs [*qpty1, qpty2, qkwp7*] were novel and could be specific for 93-11 and Nipponbare. Three QTLs [*qkwp8, qnok8 and qpht3*] were reported in the Gramene/Q-TARO database without gene designations (Table S5). Of the seven QTLs detected for DTH, the alleles from Nipponbare decreased DTH at four loci while three alleles increased DTH. All seven QTLs for DTH had been reported previously (Table S5). The similarity of the regions associated with QTLs in this study for DTH compared to other studies involving *indica* and *japonica* cultivars suggests that the same alleles are responsible for DTH across different genetic and environmental backgrounds. Of the nine QTLs detected for PHT, Nipponbare alleles increased PHT at three QTLs. All nine QTLs for PHT had been previously reported (Table S5). All the QTLs for NOT had been reported earlier (Table S5). In addition, seven QTLs for NOT, TGW and PLE, and six QTLs for grain yield per plant were reported in multiple rice germplasm lines in rice production areas worldwide. These findings suggest that these QTLs may be intensively selected during the domestication and breeding process.

In comparison with parents, changes including increased DTH, increased PHT, narrow PTY, increased NOT, slightly decreased PLE, moderate NOB, decreased number of NOK, decreased KWP, moderate TGW and decreased TYP were observed in the F_1_ hybrid (Table S1). The *OsSUT1* gene at QTL [*qdth1*] was located on chromosome 3 for DTH and PHT [64]. Another QTL, *Ghd7* on chromosome 7 was predicted to encode a CCT-domain protein controlling grain yield, PHT and DTH in rice [15]. For PTY, the *TAC1*gene on chromosome 9 was identified at *qTA-9* in a 93-11xNipponbare cross [5]. The cytokinin dehydrogenase 5 precursor gene [*Gn1a*, Os01g56810] for grain number was located at *qple1* for PLE [1]. The *OsSPL14* gene encoding a squamosa promoter-binding-like protein 9 for panicle number, panicle branching and high grain productivity was located at *qnob4* [64]. The *Hd1* gene [zinc finger protein CONSTANS] for DTH was located at *qdth5* [12]. The *TAC1*gene for PTY was located at *qpty3* [65] (Table S9).

Consistently, In F1 hybrid, HPL and LPL may explain dominance and AHPL and BLPL may explain over-dominance (Table S4). For example, genes for the AHPL in F1 are involved in plant growth, development and signal transduction including granule-bound starch synthase I, growth regulator and phosphatidylinositol 3- and 4-kinase family protein. The genes involved in carbon fixation and photosynthesis pathways were the AHPL expression pattern in F_1_ leaf including vacuolar ATP synthase subunit D 1, vacuolar ATP synthase catalytic subunit A, ribulose-phosphate 3-epimerase, uridine/cytidine kinase-like 1, ribose-5-phosphate isomerase, ferredoxin and ferredoxin-NADP reductase. Genes involved in sucrose biosynthesis phosphoglucomutase and sucrose-phosphate synthase 1 belonged to AHPL (Table S6). Similarly, genes for photosynthesis, carbon fixation, starch and sucrose metabolism were mapped at yield related QTL, and their enhanced expressions were found in the super rice hybrid [4]. Besides photosynthesis, and sucrose and starch pathways, the oxidative phosphorylation, citrate cycle [TCA cycle], and stress-resistant pathway, etc., may also contribute to heterosis [46]. In our study, the gene for sucrose phosphate synthase [SPS], the major limiting enzyme for sucrose synthesis, mapped at *ph1* responsible for plant height, and was highly expressed [AHPL] in F_1_ hybrid leaves compared to that of the parents (Table S6). The higher SPS activity was proposed to be responsible for increasing panicle length [9].

Heterosis may also be a combination of genetic and epigenetic regulation [26], [33], [43]. Altered gene expression caused by interactions between transcription factors and the allelic promoter region in the hybrids was one plausible mechanism for heterosis in rice [50]. Many differentially expressed TF genes in super hybrid rice were located in grain yield related QTLs [43]. In this study, the TF genes belonging to helix-loop-helix DNA binding domain containing protein genes of TF family HLH including Os03g53020 [*qdth2, qnot4*], Os01g57580 [*qple1*] and Os02g47660 [*qple2*] showed the AHPL expression pattern in leaves, roots and meristems, and located in the mapped yield related QTL. The *LAX1* gene encoding a bHLH transcription factor was involved in the formation of all types of axillary meristems throughout the ontogeny of rice [66] and a mutant of LAX1 [lax 1–2] was shown to reduce tiller number [67] suggesting that LAX1 function may be required for the generation of axillary meristems of both tillers and panicles [67].

The TF family AP2-EREBP members were potential targets of miRNA [68]. Noncoding RNAs were involved in epigenetic regulations, and other epigenetic mechanisms including DNA methylation, acetylation and deacetylation of histones, and chromatin remodeling [69]–[71]. Three genes encoding AP2 domain containing protein belonging to TF family AP2-EREBP showed an AHPL expression pattern in F_1_ hybrid leaves (Table S6). In *Arabidopsis* epigenetic regulation of a few regulatory genes for growth and development were observed in hybrids [55]. In this study, a total of 99 chromatin-related genes were expressed in leaves, roots and meristem tissues. Specifically, several epigenetic related genes belonging to flowering, histone demethylases, histone deacetylases, argonaute like protein genes, and polycomb group genes were highly expressed F_1_ hybrids suggesting their potential roles heterosis.

In the past decade, oligoarrays, SAGE, MPSS, and SBS have been used for transcriptome profiling. Illumina's MPSS technology has been used to generate expression data for many organisms [58], [59], [77]–[79]. Thus far, MPSS was the most popular tag based technology for sequencing of the transcriptomes of various organisms [72]–[75], [76]–[78]. More genes were identified using MPSS technology than SAGE or oligoarrays [76]. In this study, MPSS technology was used to analyze the transcriptomes of the leaves, roots and meristem tissues obtained from Nipponbare, 93-11 and their F_1_ hybrid. A total of 1 to 3 million signatures were obtained from each library. The number of redundant and non-redundant signatures generated in this study was similar to those in previous reports in rice and *Arabidopsis* [58], [59], [72]–[76]. It is important to note that significant proportion of MPSS signatures failed to match Nipponbare genome. One of the most plausible explanations is alternate splicing. Published reports demonstrated that alternative splicing in rice ranged from 13 to 21% [79]. Other possibilities also include sequencing errors in the MPSS signatures and in the Nipponbare genome or un-sequenced regions of the Nipponbare genome and also unknown mechanisms.

In summary, MPSS technology was used to obtain genome wide expression profiles in leaves, roots and meristem from ‘Nipponbare’ and ‘93-11’, and their F1 hybrid. Commonly and specifically expressed rice genes were identified, and mapped to 71 yield related QTL regions for days to heading, plant height, plant type, number of tillers, main panicle length, number of primary branches per main panicle, number of kernels per main panicle, total kernel weight per main panicle, 1000 grain weight and total grain yield per plant. Differentially expressed genes at yield related QTLs are the important candidate genes for further functional validation to unravel their role in positive and negative heterosis in F1 hybrids. This study provides the starting genomic materials to elucidate the molecular basis of yield related traits and heterosis in rice.

## Materials and Methods

The cross between *japonica* cultivar Nipponbare and *indica* cultivar 93-11 was made at Ohio State University. The RIL population an F_5-7_ was developed using an F_2_ and F_3_ single seed decent method under greenhouse conditions at the USDA Agricultural Research Service Dale Bumpers National Rice Research Center [DB NRRC].

### Phenotype evaluation

Phenotypes of RILs and their parents were evaluated in two locations in a randomized complete block design with three replications in the fields at the DBNRRC, Stuttgart, Arkansas and Rice Research Unit [RRU], Beaumont, Texas in 2009, 2010, 2011 and 2012. Population of 257, 254, 205 and 231 RILs were planted in the field at the DBNRRC, Stuttgart, Arkansas in 2009 [Stuttgart-2009, AR], 2010 [Stuttgart-2010, AR], 2011 [Stuttgart-2011, AR] and 2012 [Stuttgart-2012, AR], respectively. A planting represented by 36 plants with 20 cm spacing was performed at Stuttgart for the years 2009 and 2010. In 2011 and 2012 at Stuttgart, a planting was represented by 3 plants in a row [2 meter single rows], 61 cm alleys, and 61 cm row spacing was maintained. Similarly, a subset of 82 and 252 RILs was planted in the field of RRU, Beaumont, Texas in 2009 [Beaumont-2009, TX] and 2010 [Beaumont-2010, TX], respectively, represented by 12 plants with 20 cm spacing. For the Stuttgart and Beaumont locations in 2009 and 2010, three central plants per RIL were marked and the main panicle of each marked plant was tagged at the heading stage. These marked plants were representative of the corresponding RIL. The panicles of each marked plant were harvested individually for characterization of yield components. For the Stuttgart location in 2011 and 2012, all three plants representing each RIL were tagged at the heading stage and subsequently harvested to measure yield components (Table 1). The trait data collected per location varied depending on the weather conditions during that growing season [ex: hot summer]. The traits DTH, PHT and PTY were obtained in six field experiments. The KWP, LOG, NOT, NOK, PLE, TGW and TYP were obtained from four field experiments. NOB was obtained in three field experiments (Table 1).

### Evaluation of yield related traits

Yield related traits were measured in RILs using a modified procedure from Moncada et al. [80] and a source book from the International Rice Research Institute [IRRI] [November 2002] entitled ‘Standard Evaluation System for Rice’. Number of days to heading was recorded when 50% of plants had flowers on at least one panicle. Lodging was measured using 0–9 scale, where 0 stands for no lodging, 1 stands for up to 10% lodged, 2–11 to 20% lodged, 3–21 to 30% lodged, 4–31 to 40% lodged, 5–41 to 50% lodged, 6–51 to 60% lodged, 7–61 to 70% lodged, 8–71 to 80% lodged, 9–81 to 100% lodged. Tiller angle [plant type] was measured using 1–9 scale, where 1-tillers were erect with an angle less than 30° from the perpendicular; 3-tillers were intermediate -the angle was about 45°; 5-tillers were open- the angle was about 60°; 7-tillers were spreading- angle was more than 60° but the culms do not rest on the ground; 9-procumbent the culm or its lower part rests on ground surface. Plant height [cm] was measured for each plot. Averages were calculated for each RIL and each trait in all 6 experiments [mentioned above]. Number of tillers per marked plant was counted. One ‘main panicle’ from the marked plant was harvested to record panicle length [length of the panicle from the base to tip of the panicle], number of primary branches per main panicle, number of kernels per main panicle, and 1000 grain weight per main panicle. Main panicle data was collected for each marked RIL. The total grain yield per plant was calculated collecting all the kernels from the entire plant [for each marked RIL]. Phenotypic data were analyzed using Microsoft access and JMP Genomics [version 5.1] software (Table 1).

### Genotyping and data analysis

A total of 266 F_5_-F_7_ RILs were used for SSR analysis. DNA extraction and quantification was performed as previously described [81], [82]. Except for two indels and two SSR primers designed in house the primer sequences and map position of the SSR markers were obtained from the Gramene database [http://www.gramene.org/qtl/index.html] [83]–[85]. LJSSR1 and Con673 sequences were described by Li et al. [85]. MJIndel1 and MJIndel 2 were designed using the annotated Nipponbare and 93-11 genomes. The sequences for MJIndel 1 were F: attggatcaacacaccacac R: cagtcgaactccatcttcct and MJIndel 2 were F: aacttcaacaccaccctttga R: tttccaggtccagctcctaa. Marker amplification and allele calling were determined as described by Liu et al. [81]. A linkage map was constructed using JoinMap 4 based on the Kosambi function. Composite interval mapping [CIM] was used for phenotypic data obtained from 6 field experiments using Windows QTL Cartographer version 2.5 to identify QTLs affecting each yield related trait. The threshold was estimated by 1000 permutations at P <0.01 by QTL cartographer for each trait (Table S5). This LOD threshold was used to declare the presence of a putative QTL in order to compare with the previously identified yield related QTLs on rice chromosomes in the Gramene QTL database. -Known QTLs were identified in the public gramene rice QTL database [November 19^th^ 2012 release] [ftp://ftp.gramene.org/pub/gramene/CURRENT_RELEASE/data/qtl/] [82] and Japanese rice QTL database - http://qtaro.abr.affrc.go.jp/qtab/table [86].

### Plant materials for gene expression analysis and total RNA extraction

Nipponbare, 93-11 and their F_1_ hybrid were grown in a Conviron growth chamber at 70% relative humidity with 12 h of light [500 µmol photons m-2 sec-1] at 26°C followed by 12 h of dark at 20°C temperature. Plants from eight week old parents and their F_1_ hybrid were used to collect leaves, roots and meristem tissues. Total RNA was extracted separately from leaves, roots and meristem tissues from each plant using Trizol reagent [Invitrogen] according to the manufacturer's instructions.

A total of 21 MPSS libraries were constructed from leaf, root and meristem tissues obtained from Nipponbare, 93-11 and their F_1_ hybrid. The libraries included 4 replications from Nipponbare leaves [NLA, NLB, NLC, NLD], 2 replications from Nipponbare roots [NRA, NRB], 1 replication from Nipponbare meristems [NME], 4 replications from 93-11 leaves [I9LA, I9LB, I9LC, I9LD], 2 replications from 93-11 roots [I9RO, I9RR], 1 replication from 93-11 meristems [I9ME], 4 replications from F_1_ hybrid leaves [FLA, FLB, FLC, FLD], 2 replications from F_1_ hybrid roots [FRO, FRR], and 1 replication from F_1_ hybrid meristems [FME]. Each replication represented 4 individual plants. Since MPSS technology was very expensive, we restricted the number of replications to 2 and 1 in root and meristem tissues, respectively.

### Construction of the MPSS libraries, sequencing, and bioinformatics

MPSS library construction, sequencing and annotation were performed essentially as previously described [58], [59], [72]–[76]. Briefly, to get high quality data, sequences obtained from MPSS technology were passed through two filters – reliability and significance. The ‘reliability’ filter determines if the given signature is found in more than one library (reliable signatures) or present in only one library (unreliable signature). The ‘significance’ filter determines if a given signature is found in any library at ≥4TPM (transcripts per million) (significant signature) or <4TPM (non-significant signature) in a normalized library. The significant and reliable distinct signatures were identified in 21 MPSS libraries as previously described. For leaf tissue, the signature frequencies data obtained from 4 replications were used to calculate the mean value. This included the transcripts expressed either in only one, two, three or all four replications. Similarly, mean values were calculated for 2 replications of root tissues. The expression levels of Nipponbare and 93-11 were compared with that of their F_1_ hybrid and the differentially expressed signatures were classified into 8 expression patterns [AHPL, HPL, MPL, LPL, BLPL, SEF_1_, EOPF_1_ and EPAF_1_] with some modifications [26], [44]. To avoid sequence bias we performed cluster analysis on the signatures matching to both Nipponbare and 93-11 genomes to identify commonly and specifically expressed genes among Nipponbare, 93-11 and F_1_ hybrid. Clustering analysis was carried out using Microsoft Access and JMP Genomics [version 5.1] software to identify the genes specifically and commonly expressed in Nipponbare, 93-11 and their F_1_ hybrid. Bioinformatic analyses including identification of antisense transcripts, alternate transcripts, TFs and functional classification of genes using KEGG database were conducted as previously described [58], [59], [72]–[76]. The entire dataset is available at the NCBI's Gene Expression Omnibus database.

## Supporting Information

Figure S1 **Transgressive variation among the F_6–8_ generation RILs of Nipponbare X 93–11 cross in the field at Stuttgart, Arkansas.** Also, phenotypic variation (plant height, number of tillers and maturity) of F_1_ hybrids and their parents in both field and growth chamber conditions.
(TIF)

Figure S2 Filter results for 21 MPSS libraries. **A total of 179,151 distinct 17-base expressed signatures from 21 MPSS libraries were processed according to three filters- significance, reliability, and genomic match as described by Meyers et al.** **[58]**–**[59]**.
(TIF)

Figure S3 ‘**Kyoto Encyclopedia of Genes and Genomes’ based functional classification of genes induced in F_1_ hybrid (leaves, roots and meristem tissues) compared to their parents Nipponbare and 93–11.** Genes showing expression level pattern AHPL were only used for this analysis.
(TIF)

Table S1 **Phenotypic variation for yield related traits among the RILs in six field studies (Stuttgart-2009, AR; Stuttgart-2010, AR; Stuttgart-2011, AR; Stuttgart-2012, AR; Beaumont-2009, TX and Beaumont-2010, TX).** Phenotypic variation was observed for days to heading, plant height, tiller angle, tiller number, panicle length, number of primary branches per panicle, number of kernels per panicle, total kernel weight per panicle, 1000 grain weight and total grain yield per plant.
(XLSX)

Table S2 **Characteristics of MPSS libraries.** Library statistics of Nipponbare, 93-11 and F_1_ hybrid from leaves, roots and meristem tissues.
(DOCX)

Table S3 **Classification of the MPSS signatures based on their location on the annotated gene (hits = 1) (See Meyers et al.** [58]–[59] **for details).** Reliable and significant (≥4TPM) MPSS signatures obtained from leaf, root and meristem libraries (mean of replications in each tissue) of Nipponbare, 93–11 and their F1 hybrid are summarized.
(DOCX)

Table S4 **Gene expression levels in leaf, root and meristem tissues of F_1_ hybrids and their parents Nipponbare and 93–11.** Mean signature frequencies (copy number) were calculated from four leaf replications and two root replications separately. The mean signature value of F_1_ hybrids was compared with their parents to classify the signature into one of the 8 expression patterns (AHPL, HPL, MPL, LPL, BLPL, SEF1, EOPF_1_ and EPAF_1_). Detailed annotation of each signature is presented.
(XLSX)

Table S5 **List of known and novel yield related QTL identified in this study based on Gramene QTL database (** ftp://ftp.gramene.org/pub/gramene/CURRENT_RELEASE/data/qtl/ **) and Japanese rice QTL database (Q-TARO database) -** http://qtaro.abr.affrc.go.jp/qtab/table **.** SSR marker intervals, percent variation, additive effect and LOD score for each QTL identified in each location is presented. Average LOD score was calculated for QTL identified in more than one location. Similar QTL locations identified in other studies (indicated in RED font color) are presented for the known QTL (in our study). Yellow highlighted QTL are reported in Japanese Q-TARO database.
(XLSX)

Table S6 **List of all expressed genes identified in mapped yield related QTL regions.** The expressed genes located between the two flanking SSR markers of yield related QTL are listed. Also, the expressed genes belonging to SEF1 and AHPL expression pattern categories located in mapped yield related QTL regions are also listed. The QTL identified by mean LOD score ≥5 are used for this analysis.
(XLSX)

Table S7 **List of TF genes identified in mapped yield related QTL regions.** The TF genes located between the two flanking SSR markers of yield related QTL are listed. The QTL identified by mean LOD score ≥5 are used for this analysis. TF family names and the expression level patterns of TF genes in different tissues are presented here.
(XLSX)

Table S8 **List of chromatin related genes identified in mapped yield related QTL regions.** The chromatin related genes located between the two flanking SSR markers of yield related QTL are listed. The QTL identified by mean LOD score ≥5 are used for this analysis.
(XLSX)

Table S9 **Cloned yield related genes located in mapped QTL regions in our study.** The genes responsible for yield related traits have been isolated in different studies and the information is deposited in Japanese Q-TARO database (http://qtaro.abr.affrc.go.jp/ogro/table).
(XLSX)
