# Supplementary figures and images for: Identification of Candidate Genes Associated with Positive and Negative Heterosis in Rice
Source: PLoS One. 2014 Apr 17;9(4):e95178. doi: 10.1371/journal.pone.0095178 (PMC3990613; doi:10.1371/journal.pone.0095178)

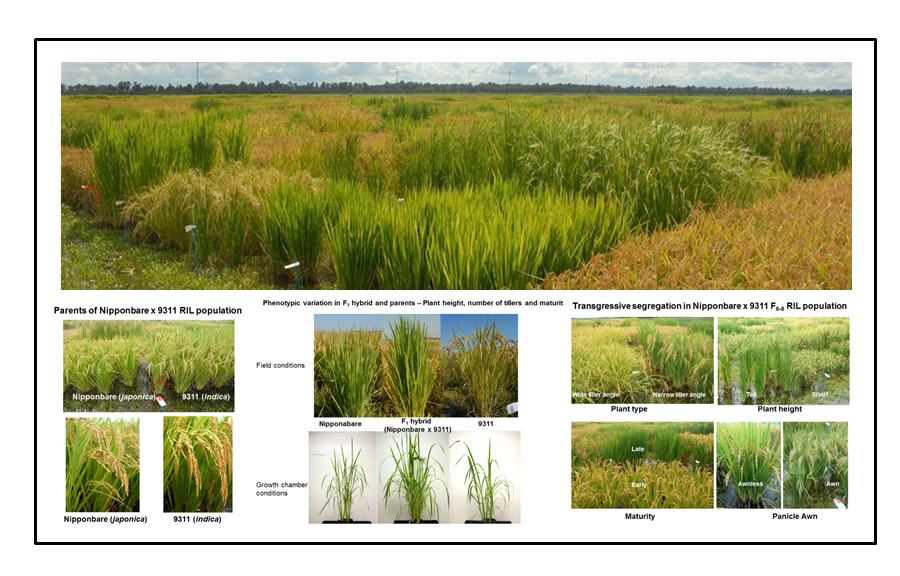

Supplement: Figure S1 — Transgressive variation among the F6–8 generation RILs of Nipponbare X 93–11 cross in the field at Stuttgart, Arkansas. Also, phenotypic variation (plant height, number of tillers and maturity) of F1 hybrids and their parents in both field and growth chamber conditions. (TIF) [file pone.0095178.s001.tif]

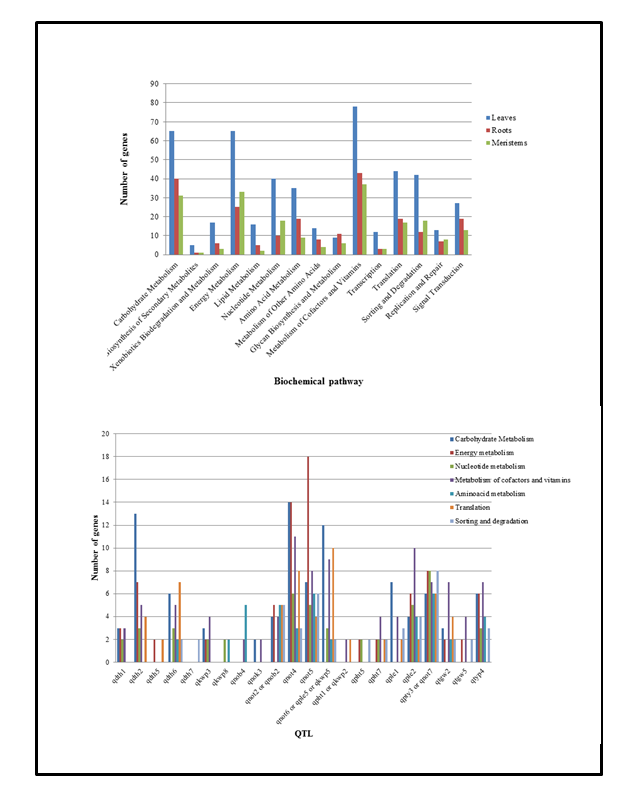

Supplement: Figure S3 — ‘Kyoto Encyclopedia of Genes and Genomes’ based functional classification of genes induced in F1 hybrid (leaves, roots and meristem tissues) compared to their parents Nipponbare and 93–11. Genes showing expression level pattern AHPL were only used for this analysis. (TIF) [file pone.0095178.s003.tif]
