# Supplementary material for: Identification of Candidate Genes Associated with Positive and Negative Heterosis in Rice
Source: PLoS One. 2014 Apr 17;9(4):e95178. doi: 10.1371/journal.pone.0095178 (PMC3990613; doi:10.1371/journal.pone.0095178)
Supplement: Table S2 — Characteristics of MPSS libraries. Library statistics of Nipponbare, 93-11 and F1 hybrid from leaves, roots and meristem tissues. (DOCX) [file pone.0095178.s005.docx]

**Table S2.** **Characteristics of MPSS libraries.**

|  | NLA | NLB | NLC | NLD | Avg_NPB leaves | NRA | NRB | Avg_NPB roots | NME | I9LA | I9LB | I9LC | I9LD | Avg_9311 leaves | I9RO | I9RR | Avg_9311 roots | I9ME | FLA | FLB | FLC | FLD | Avg_ F1 hybrid leaves | FRO | FRR | Avg_ F1 hybrid roots | FME |
| --- | --- | --- | --- | --- | --- | --- | --- | --- | --- | --- | --- | --- | --- | --- | --- | --- | --- | --- | --- | --- | --- | --- | --- | --- | --- | --- | --- |
| Total reads sequenced | 1,073,991 | 1,348,557 | 1,263,549 | 1,254,824 | 4940921 | 2,675,567 | 2,617,770 | 5293337 | 2,568,641 | 1,606,175 | 1,005,937 | 1,144,192 | 1,146,212 | 4902516 | 2,162,940 | 2,156,164 | 4319104 | 2,112,790 | 1,171,478 | 1,040,468 | 1,056,621 | 1,419,115 | 4687682 | 2,436,387 | 2,205,884 | 4642271 | 3,045,290 |
| Distinct | 26461 | 24612 | 17394 | 20791 | 60782 | 31163 | 27908 | 48206 | 28621 | 15089 | 17546 | 15901 | 16344 | 38478 | 24026 | 19087 | 34771 | 21836 | 14127 | 21004 | 18884 | 20326 | 44455 | 23174 | 21908 | 29119 | 24715 |
| Reliable* | 18917 | 20549 | 15322 | 18659 | 44971 | 26594 | 22335 | 38064 | 24183 | 13507 | 15978 | 13949 | 14664 | 31696 | 19386 | 17065 | 28109 | 17570 | 12544 | 19167 | 17466 | 18791 | 38082 | 20804 | 19450 | 24291 | 19945 |
| Significant* | 21670 | 20998 | 15759 | 18690 | 48688 | 26608 | 22554 | 38330 | 24305 | 13736 | 16606 | 14339 | 14997 | 33332 | 20162 | 17360 | 29195 | 19027 | 13146 | 19780 | 17976 | 19001 | 40037 | 20636 | 19243 | 24476 | 20056 |
| Reliable and Significant (≥4TPM) | 16405 | 16035 | 13319 | 14395 | 35617 | 19124 | 16732 | 27282 | 18106 | 11578 | 14290 | 11910 | 12519 | 26697 | 16129 | 14078 | 22996 | 14081 | 10726 | 17098 | 15436 | 15822 | 32370 | 15407 | 14601 | 17289 | 15033 |
| 1-100 TPM | 24033 | 21839 | 14767 | 18132 | 57321 | 28741 | 25148 | 45257 | 26237 | 12121 | 14781 | 13218 | 13661 | 35346 | 21009 | 16296 | 31371 | 19695 | 11805 | 18005 | 16058 | 17724 | 41236 | 21126 | 19747 | 26983 | 22579 |
| 101-1,000 TPM | 2248 | 2610 | 2475 | 2497 | 3279 | 2250 | 2611 | 2804 | 2245 | 2801 | 2596 | 2504 | 2501 | 2973 | 2853 | 2613 | 3249 | 1962 | 2081 | 2847 | 2673 | 2454 | 3072 | 1860 | 1958 | 1958 | 1950 |
| 1,001-10,000 TPM | 172 | 156 | 146 | 153 | 190 | 167 | 146 | 151 | 134 | 159 | 163 | 172 | 175 | 169 | 161 | 173 | 158 | 166 | 232 | 148 | 147 | 141 | 161 | 179 | 193 | 184 | 176 |
| >10,000 TPM | 8 | 7 | 6 | 9 | 8 | 5 | 3 | 4 | 5 | 8 | 6 | 7 | 7 | 6 | 3 | 5 | 3 | 13 | 9 | 4 | 6 | 7 | 7 | 9 | 10 | 10 | 10 |
| #Distinct genes | 5964 | 7984 | 7562 | 8146 | 11902 | 9420 | 8670 | 11816 | 9360 | 6207 | 7701 | 6485 | 6710 | 10890 | 8014 | 7435 | 10186 | 6696 | 5444 | 9037 | 8422 | 8641 | 12717 | 7747 | 7299 | 8007 | 7591 |
| $ Genome match | 9224 (56%) | 12152 (76%) | 11416 (86%) | 12546 (87%) | 22786 (64%) | 15660 (82%) | 13200 (79%) | 21122 (77%) | 14936 (83%) | 9582 (83%) | 12414 (87%) | 9967 (84%) | 10463 (84%) | 20991 (79%) | 12734 (79%) | 11564 (82%) | 17756 (77%) | 10777 (77%) | 8073 (75%)  ***8147 (76%)*** | 14124 (83%)  ***14241 (83%)*** | 12877 (83%)  ***12993 (84%)*** | 13346 (84%)  ***13384 (85%)*** | 24717  76%)  ***24895 (77%)*** | 12278  (80%)  ***12419 (81%)*** | 11322  (78%)  ***11453(78%)*** | 12855  (74%)  ***13005 (75%)*** | 11763  (78%)  ***11857***  ***79%)*** |

Note: *- Number of signatures passed through reliability and significance filters as described by Meyers et al. 2004

#- Using all reliable and significance filter signatures

$- Number and % of signatures match Nipponbare /93-11 /both genomic sequences (≥4 TPM)

*Italics*- indicate match to 93-11 genome
