# Supplementary material for: Identification of Candidate Genes Associated with Positive and Negative Heterosis in Rice
Source: PLoS One. 2014 Apr 17;9(4):e95178. doi: 10.1371/journal.pone.0095178 (PMC3990613; doi:10.1371/journal.pone.0095178)
Supplement: Table S3 — Classification of the MPSS signatures based on their location on the annotated gene (hits = 1) (See Meyers et al. [58]–[59] for details). Reliable and significant (≥4TPM) MPSS signatures obtained from leaf, root and meristem libraries (mean of replications in each tissue) of Nipponbare, 93–11 and their F1 hybrid are summarized. (DOCX) [file pone.0095178.s006.docx]

**Table S3**. **Classification of the MPSS signatures based on their location on the annotated gene (hits=1) (See Meyers et al. [40-42] for details).**

| MPSS Signature category | Nipponbare | | | | | | 93-11 | | | | | | F1-Hybrid | | | | | |
| --- | --- | --- | --- | --- | --- | --- | --- | --- | --- | --- | --- | --- | --- | --- | --- | --- | --- | --- |
|  | Leaves | | Roots | | Meristems | | Leaves | | Roots | | Meristems | | Leaves | | Roots | | Meristems | |
|  | Total signatures | *Grouped by gene | Total signatures | *Grouped by gene | Total signatures | *Grouped by gene | Total signatures | *Grouped by gene | Total signatures | *Grouped by gene | Total signatures | *Grouped by gene | Total signatures | *Grouped by gene | Total signatures | *Grouped by gene | Total signatures | *Grouped by gene |
| Class 1 | 8108 | 6731 | 7426 | 6402 | 5339 | 4874 | 7296 | 6123 | 6136 | 5424 | 4027 | 3663 | 8615 | 7128 | 5301 | 4654 | 4711 | 4267 |
| Class 2 | 6180 | 5421 | 6287 | 5575 | 4655 | 4255 | 5510 | 4895 | 5204 | 4719 | 2930 | 2750 | 6905 | 5965 | 3241 | 3013 | 3378 | 3133 |
| Class 3 | 2367 | 2102 | 2255 | 2019 | 1398 | 1304 | 2136 | 1894 | 1701 | 1541 | 943 | 887 | 2801 | 2438 | 1359 | 1260 | 1043 | 960 |
| Class 4 | 1381 | 0 | 1073 | 0 | 759 | 0 | 1014 | 0 | 758 | 0 | 476 | 0 | 1435 | 0 | 580 | 0 | 544 | 0 |
| Class 5 | 690 | 641 | 502 | 470 | 319 | 311 | 494 | 465 | 380 | 355 | 207 | 200 | 812 | 751 | 235 | 227 | 230 | 223 |
| Class 6 | 218 | 210 | 187 | 176 | 123 | 119 | 190 | 185 | 141 | 138 | 65 | 64 | 240 | 234 | 88 | 85 | 70 | 68 |
| Class 7 | 231 | 227 | 203 | 202 | 141 | 141 | 217 | 213 | 173 | 171 | 96 | 96 | 261 | 256 | 127 | 127 | 114 | 114 |
| Classes 1,2,5,7 | 15209 | 11065 | 14418 | 11068 | 10454 | 8720 | 13517 | 10127 | 11893 | 9507 | 7260 | 6203 | 16593 | 11889 | 8904 | 7327 | 8433 | 8428 |
| Classes 3,6 | 2585 | 2584 | 2442 | 2169 | 1521 | 1418 | 2326 | 2050 | 1842 | 1664 | 1008 | 949 | 3041 | 2640 | 1447 | 1337 | 1113 | 1020 |
| Total | 19175 | 11902 | 17933 | 11816 | 12734 | 9360 | 16857 | 10890 | 14493 | 10186 | 8744 | 6696 | 21069 | 12717 | 10931 | 8007 | 10090 | 7591 |

Note: *- Grouped by gene including transposons

Class 1 - Exon, sense strand

Class 2 - 500 bp 3’-UTR

Class 3 - Exon, antisense strand

Class 4 - Un-annotated region

Class 5 - Intron, sense strand

Class 6 - Intron, antisense strand

Class 7 - Span splice site, sense strand

Classes 1,2,5,7 - Sense signatures

Classes 3,6 - Antisense signatures
